# Functional Immunopeptidomics by Characterizing Immunopeptides Against HLAs, TCRs, and Antibodies

**DOI:** 10.21203/rs.3.rs-11040221/v1

**Published:** 2026-10-08

**Authors:** Hongyi Liu, Effram Wei, Yingwei Hu, Yuefan Wang, Zhenyu Sun, Hui Zhang

**Affiliations:** 1. Department of Pathology, Johns Hopkins University School of Medicine, Baltimore, MD 21231, USA; 2. Department of Chemical and Biomolecular Engineering, Johns Hopkins University, Baltimore, MD 21218, USA

**Keywords:** HLA, immunopeptidomics, TCR, antibody, glycosylation, phosphorylation, RCC

## Abstract

Target-directed immunotherapies, including TCR-T cell therapies, targeted antibody therapy, and therapeutic vaccines, rely on the identification of tumor-specific antigens (TSAs). However, classical immunopeptidomics is severely constrained by an inability to distinguish passively presented self-ligands of HLAs from functionally recognized by T cells. To resolve these challenges, we developed immunopeptidomics for receptor-associated antigens, a platform capable of sequentially isolating immunopeptidome and their functions with immune cell receptors from a single clinical tissue specimen. Applied to a cohort of 30 renal cell carcinoma (RCC) specimens (18 primary tumors and 12 paired normal adjacent tissues), this approach generated expansive presentation maps encompassing 38,848 unique HLA-I and 26,961 unique HLA-II peptides. Functional receptor captures (IgG- and TCR-bound) achieved up to 5.3-fold higher tumor-specific peptide (TSP) purity ratios compared to direct baseline HLA isolation. Furthermore, cross-fractional co-identification validated recurrent, high-priority unmutated targets (NNMT, TMBIM6, RPL3, VIM, and ENPP3) alongside tumor-specific phosphopeptide hubs and macrophage-restricted N-glycopeptides (CD68). Integrating companion global proteomics uncovered a fundamental regulatory divergence between MHC classes: HLA-I presentation throughput scales linearly (R = 0.883) via a STAT1-orchestrated transcriptional program driving the antigen presentation machinery (APM), whereas HLA-II presentation is uncoupled from parental protein abundance (R = 0.302) due to asynchronous downstream endolysosomal processing and proteolytic over-cleavage kinetics. Together, this platform establishes a micro-input framework that unifies physical presentation with native functional characterization and microenvironmental regulation to accelerate target discovery in low-TMB human cancers.

## INTRODUCTION

The dawn of personalized cancer immunotherapy epitomized by T-cell receptor engineered T-cell (TCR-T) therapies, targeted antibody therapy, and therapeutic vaccines has fundamentally shifted the oncology paradigm toward the precise eradication of malignant clones^1–3^. The clinical efficacy of these modalities hinges on the presentation of human leukocyte antigen (HLA)-restricted peptides that possess high tumor specificity and immunogenicity^4–6^. For over a decade, genomics-guided target pipelines have focused almost exclusively on somatic mutational neoantigens^7–9^. However, in a vast landscape of human malignancies characterized by a low tumor mutational burden (low-TMB), such as renal cell carcinoma (RCC), computationally predicted single-nucleotide mutational neoantigens are exceedingly scarce and rarely achieve actual cell-surface presentation^10–12^. Despite its low TMB, RCC exhibits profound susceptibility to immune checkpoint blockade^13–15^, a paradox indicating that the true driving force of anti-tumor immunity in these malignancies is mediated by an unmasked reservoir of non-mutational tumor-specific antigens (TSAs)^16,17^.

Beyond unmutated protein overexpression, aberrant post-translational modifications (PTMs), which most notably include phosphorylation and glycosylation, have emerged as a pivotal yet under-explored frontier of non-canonical TSAs^18,19^. Malignant transformation is universally accompanied by hyper-activated oncogenic kinase cascades and dysregulated glycosylation pathways^20–22^. These alterations generate unique structural neo-epitopes that are absent in normal tissues, directly expanding the functional immunopeptidome^23–25^. Phosphorylated HLA ligands, often derived from critical signal transducers and oncogenes, can escape central tolerance while exhibiting altered susceptibility to endolysosomal degradation^26^. Concurrently, cell-surface and microenvironmental glycopeptides reflect the specialized activation and oncogenic remodeling of infiltrating immune sub-populations^27,28^. However, systematically mapping these modified immunopeptides from clinical specimens has remained extraordinarily challenging due to their substoichiometric abundance, ionization suppression, and the lack of specialized low-input extraction workflows^29^.

Direct mass spectrometry (MS)-based immunopeptidomics has emerged as a powerful analytical tool for identifying the physical presentation of these hidden target landscapes^30,31^. Yet, classical immunopeptidomics protocols remain bottlenecked by material requirements^32–35^. Standard liquid chromatography-tandem mass spectrometry (LC-MS/MS) captures passive “passenger” HLA ligands without determining active recognition by endogenous tumor-infiltrating lymphocytes (TILs)^36,37^. Consequently, there is an urgent technological demand for platforms capable of simultaneously mapping physical HLA presentation, post-translational modifications, and direct functional receptor reactivity^38^.

To overcome these long-standing methodological constraints, we developed sequential micro-scale immunopeptidomics for receptor-associated antigens, a fluidic platform capable of extracting functionally enriched immunopeptidome architectures from a mere 10 mg of cryopulverized clinical tissue. Operating as a continuous fluidic cascade, this platform sequentially captures endogenous IgG, HLA II, HLA I, and T-cell receptors (TCRs) from a single lysate, integrating an *in vitro* equilibration binding assay to isolate specific, functionally active receptor-bound ligands without relying on speculative computational predictions. Beyond establishing micro-scale technical feasibility, deploying this platform across a clinical RCC cohort reveals fundamental biological insights into the governance of the tumor immunopeptidome. Here, we demonstrate that the platform successfully maps recurrent unmutated public targets (NNMT, TMBIM6, RPL3, VIM, ENPP3) alongside an extensive landscape of tumor-specific phosphopeptides and macrophage-restricted CD68 N-glycopeptides. Crucially, by integrating companion global proteomics and Gene Set Enrichment Analysis (GSEA), we uncover a fundamental mechanistic divergence between MHC classes: HLA I presentation throughput is tightly coupled to a STAT1-mediated transcriptional scaling of the antigen presentation machinery (APM) in immune-hot tumors, whereas HLA-II presentation is uncoupled due to fractured and asynchronous downstream endolysosomal processing modules. Together, this work establishes a robust, low-input framework that bridges physical presentation, PTM diversity, native functional characterization, and microenvironmental regulation to accelerate targeted immunotherapy discovery.

## RESULT

### Profiling of Functional HLA II-associated global, phosphorylated, and glycosylated immunopeptides in renal cell carcinoma

To circumvent the long-standing limitations of conventional immunopeptidomics, namely, the requirement of high tissue inputs and the absence of functional, receptor-engagement contexts, we developed a sequential immunoaffinity extraction and reconstitution platform (Supplementary Figure 1). Utilizing 10 mg of cryopulverized renal cell carcinoma (RCC) tumor tissue, the lysate is directed through a path consisting of four consecutive parts: (1) native IgG enrichment (native IgG binding HLA peptides, nIgG), (2) HLA-II enrichment, (3) HLA-I enrichment, and (4) T-cell receptor (native TCR binding HLA peptides, nTCR) enrichment. Unbound flowthroughs from each stage serve directly as the entry material for the subsequent tier. Crucially, to capture the distinct subpopulation of HLA ligands actively engaged by the local immune repertoire, an in vitro reconstitution binding assay was integrated. In this step, a portion of the isolated HLA-I and HLA-II peptides are incubated with the neutralized, bead-bound native IgG or TCR proteins captured from the same specimen, yielding receptor-bound immunopeptide fractions designated as bIgG and bTCR, respectively. All generated fractions are subsequently characterized via high-resolution LC-MS/MS analysis.

We evaluated the analytical capacity of this platform across a cohort of 30 RCC tissue specimens, including 18 primary tumor tissues and 12 paired normal adjacent tissues (NATs). Focusing first on the HLA II presentation landscape, the workflow identified a total of 26,961 unique peptides across all HLA II enrichment groups (Figure 1A). Dissection into discrete operational tiers identified 20,558 unique peptides in the baseline HLA II group, 2,475 in the native IgG (nIgG) group, and 1,832 in the native TCR (nTCR) group. Direct in vitro reconstitution isolated 7,609 unique peptides bound to IgG (bIgG) and 4,538 bound to TCR (bTCR) (Figure 1A). Structural characterization revealed a consistent peptide length distribution spanning 14 to 16 amino acids across all enrichment groups, matching canonical open-groove MHC Class II binding (Figure 1B). UpSet matrix analysis demonstrated 15,693 peptides strictly exclusive to baseline HLA II capture, alongside distinct intersection modules shared across antibody- and TCR-engaged cohorts (Figure 1C). Unsupervised GibbsCluster analysis confirmed high sequence purity, maintaining ~96.3% motif alignment and a low mean outlier rate of ~3.7% across patient samples (Figure 1D).

Profiling post-translationally modified immunopeptides revealed a phosphosite composition dominated by phosphoserine (52.4%, n=5,204), followed by phosphothreonine (36.2%, n=3,598) and phosphotyrosine (11.4%, n=1,127) (Figure 1E). Length distribution profiles of unique phosphopeptides remained consistent across enrichment groups (Figure 1F), with spatial mapping showing a uniform distribution along the peptide backbone from the N- to C-terminus (Figure 1G). Quantitatively, TCR-based capture modalities markedly elevated phosphopeptide recovery, with nTCR yielding 4,495 PSMs and 3,640 unique phosphopeptides (Figure 1H). For N-glycopeptides, 14 unique sequences were identified in baseline HLA II, with 2 unique glycopeptides each recovered in the nTCR and bTCR groups (Figure 1I). Glyco-PSMs displayed a length peak centered at 16 amino acids (Figure 1J) and localized glycosylation site enrichment near the C-terminus (Figure 1K). Structural glycoform profiling demonstrated that while nTCR and bTCR captured exclusively Truncated/Paucimannose structures, baseline HLA II preserved diverse complex, sialylated, hybrid, and high-mannose N-glycans (Figure 1L).

Together, these findings demonstrate that the sequential immunoaffinity extraction and reconstitution platform effectively overcomes key limitations of conventional immunopeptidomics, enabling deep, high-purity profiling of baseline and receptor-engaged HLA II landscapes from minimal tissue inputs. By simultaneously capturing functional receptor-bound sub-populations and post-translationally modified ligands, this multi-tier architecture provides a comprehensive framework to map the functional immunopeptidome.

### Characterization of HLA II-restricted tumor-specific peptides, phosphorylated peptides, and N-glycosylated peptides in renal cell carcinoma

Having established the platform’s analytical capacity, we next sought to identify clinically relevant tumor-specific peptides (TSPs) by filtering tumor immunopeptidomes against patient-matched normal adjacent tissue (NAT) controls (Figure 2). Length density profiles of HLA II peptides isolated from NAT and tumor specimens exhibited identical distributions centered at 14–16 amino acids (Figure 2A), confirming that sequential extraction preserved canonical open-groove binding structures without tissue-type bias. Assessment of peptide-spectrum match (PSM) distributions further confirmed robust presentation intensity across both tissue types (Figure 2B). Following stringent NAT subtraction, direct baseline HLA II capture yielded 8,967 unique TSPs, whereas the functional bIgG, nIgG, bTCR, and nTCR fractions recovered 3,418, 784, 1,486, and 331 unique TSPs, respectively (Figure 2C). UpSet intersection matrix analysis was performed across all 18 primary tumor specimens (Supplementary Figure 2). This revealed that while the TSP landscape is predominantly characterized by sample-exclusive singletons (e.g., up to 2,375 unique TSPs exclusive to individual tumors), discrete sub-modules of shared TSPs were consistently detected across subsets of patients spanning both immune-hot and immune-cold phenotypes (Supplementary Figure 2). To account for differences in overall baseline recovery, we evaluated normalized TSP purity ratios. Notably, receptor-based captures achieved higher median enrichment efficiency (nIgG median 1.1; nTCR median 1.3) than direct baseline HLA II capture (median 0.6) (Figure 2D). Furthermore, tumors displaying an immune-hot subtype exhibited higher TSP purity ratios compared to immune-cold tumors (Figure 2D), indicating that active immune infiltration enriches for functionally engaged tumor antigens.

Extending this subtraction framework to post-translationally modified ligands, we profiled tumor-specific phosphorylated peptides (TSPPs). TSPP presentation demonstrated substantial inter-patient heterogeneity across immune-hot and cold subtypes, with TCR capture modalities consistently outperforming IgG capture in isolating modified immunogenic epitopes (Figure 2E). UpSet intersection analysis of TSPPs and their parental source proteins across patient specimens delineated cohort-wide sharing patterns, demonstrating that while individual TSPPs were largely patientexclusive, their source proteins exhibited higher degrees of cross-patient overlap (Supplementary Figure 3). Bipartite network modeling of shared TSPPs further resolved recurrent antigen-patient connectivity, highlighting central immunogenic hubs anchored by proteins including EN2, CSMD2, FLNA, FLT4, FES, and MYO3B (Supplementary Figure 4). Cohort-wide recurrence analysis revealed shared TSPP source proteins led by high-abundance candidates such as TTN and MUC16 (Figure 2F), and a binary presence-absence matrix confirmed their recurrent presentation across multiple patient specimens (Figure 2G).

Finally, mapping tumor-specific N-glycosylated peptides to parental source proteins uncovered distinct microenvironment-dependent presentation; remarkably, CD68-derived glycopeptides were detected exclusively in immune-hot tumors (Figure 2H), aligning with increased infiltration of tumor-associated macrophages in inflamed microenvironments^39,40^. High-confidence structural assignment and sequence coverage for identified N-glycopeptides were validated by representative tandem mass spectra (MS/MS), which exhibited characteristic glycan oxonium ions, intact peptide-glycan fragment Y-ions, and complete b/y backbone ion series (Supplementary Figure 5).

Together, these findings demonstrate that combining patient-matched tissue subtraction with multi-tier receptor capture effectively isolates high-purity, recurrent tumor-specific immunopeptides, providing a targeted reservoir of post-translationally modified neoantigen candidates shaped by the tumor immune subtypes.

### Functional immunopeptidomic profile performance of HLA I-restricted peptides.

Having established the HLA II presentation landscape, we next extended our platform to interrogate HLA I-restricted immunopeptides across the RCC cohort (Figure 3). Benchmarking performance across all HLA I presentation tracks identified a total of 38,848 unique peptides across the combined dataset. Dissection into operational fractions yielded 33,487 unique peptides in baseline HLA I, 4,618 in bIgG, 5,886 in bTCR, 1,376 in nIgG, and 2,719 in nTCR (Figure 3A). In contrast to the open-groove length distribution observed for HLA II, peptide length profiling across all HLA I fractions displayed a sharp, dominant peak at 9 amino acids (9-mers), verifying structural fidelity to canonical closed-groove MHC Class I binding and confirming minimal non-specific degradation (Figure 3B). UpSet matrix analysis demonstrated 28,827 peptides strictly exclusive to baseline HLA I capture, alongside distinct multi-fractional overlap modules shared across antibody- and TCR-engaged cohorts (Figure 3C). Furthermore, unsupervised GibbsCluster deconvolution confirmed high sequence purity, maintaining >96% motif alignment and a low average outlier rate of ~3.6% across RCC specimens (Figure 3D).

To delineate tumor-specific targets within the HLA I immunopeptidome, we evaluated primary RCC tumors against patient-matched NAT controls. Length density overlays confirmed identical 9-mer binding preferences across both tissue states (Figure 3E), accompanied by robust PSM density distributions (Figure 3F). Following subtractive filtering against paired NATs, baseline HLA I capture yielded 20,118 unique TSPs, whereas the functional fractions recovered 1,840 (bIgG), 344 (nIgG), 2,451 (bTCR), and 201 (nTCR) unique TSPs (Figure 3G). To evaluate cross-patient sharing profiles of these HLA I-restricted TSPs, cohort-wide UpSet intersection analysis was conducted across all 18 primary tumor specimens (Supplementary Figure 6). Consistent with our HLA II observations, the HLA I TSP landscape demonstrated marked inter-patient heterogeneity, dominated by sample-exclusive singletons (e.g., up to 3,067 unique TSPs restricted to individual patient tumors), alongside discrete sub-modules of shared TSPs identified across subsets of both immune-hot and immune-cold tumors (Supplementary Figure 6). Crucially, evaluating normalized TSP purity ratios revealed that functional receptor captures markedly surpassed direct baseline HLA I captured in target enrichment efficiency (Figure 3H). While baseline HLA I exhibited a median purity ratio of 0.6, receptor-engaged fractions achieved substantially higher median values of 2.5 (bIgG), 5.3 (nIgG), 1.8 (bTCR), and 3.1 (nTCR) (Figure 3H). In alignment with our HLA II observations, immune-hot tumors consistently displayed higher target purity ratios than immune-cold tumors (Figure 3H), reinforcing that local immune infiltration enriches for functionally engaged HLA I ligands.

Together, these results demonstrate that the platform achieves exceptional analytical depth and motif purity for HLA I ligands, proving that multi-tier receptor capture significantly enhances tumor-specific peptide enrichment efficiency in clinically inflamed tumor microenvironments.

### Identification of functional tumor-specific immunopeptides

Having characterized the individual properties of HLA I and II presentation, we next integrated both pathways to establish a semi-quantitative, repertoire-wide map of functional tumor-specific immunopeptides across the RCC cohort (Figure 4). Quantification of analytical throughput (log scale) demonstrated that immune-hot tumors yielded higher unique peptide counts across both HLA I and II systems relative to “immune-cold” tumors (Figure 4A). Crucially, stacked fractional breakdowns revealed that beyond baseline-exclusive ligands, a substantial proportion of tumor-specific immunopeptides were co-identified across multiple operational tiers within the exact same patient specimen including dual (HLA + TCR) and triple (HLA + TCR + IgG) engagement states (Figure 4A). This co-identification demonstrates that distinct enrichment modalities independently capture identical peptide-spectrum matches (PSMs) from a single sample, providing direct, internal orthogonal validation that these specific HLA ligands are actively recognized by the local intratumoral immune repertoire.

Examining per-sample PSM distributions for post-translationally modified species further revealed individual-level presentation dynamics (Supplementary Figure 7). Across the cohort, HLA II-restricted phosphopeptide PSMs totaled 7,310 events (Supplementary Figure 7). Strikingly, sample C3N-01648 exhibited an overwhelming concentration of phosphopeptide presentation, yielding over 1,400 PSMs dominated by baseline HLA II capture (Supplementary Figure 7). Given that C3N-01648 displayed a relatively modest global/unmodified baseline HLA II peptide yield (Figure 4A), this selective hyper-presentation of phosphorylated species suggests that intense oncogenic kinase signaling or dysregulated phosphatase activity in specific tumor microenvironments can dramatically skew the immunopeptidome toward phosphopeptides, effectively compensating for lower overall baseline HLA II abundance (Supplementary Figure 7).

Granular tracking of high-priority, multi-fractional targets resolved distinct, patient-tailored presentation profiles. For HLA I, shared tumor-specific epitopes derived from proteins including NNMT (YYMIGEQKF), TMBIM6 (AYVHMVTHF), RPL3 (KFIDTTSKF), and VIM (LPLPNFSSL) displayed high PSM densities and reproducible capture within individual patient tumors (Figure 4B). Similarly, HLA II-restricted targets originating from VIM (TLLIKTVETRDG), TMBIM6 (AYVHMVTHF), and ENPP3 (VPFYEPSHAEEVSK and KVPFYEPSHAEEVSK) demonstrated robust co-identification across baseline and receptor-bound fractions (Figure 4C). Crucially, cross-referencing companion global proteomic data established that the parental source genes for these multi-engaged targets (NNMT, TMBIM6, RPL3, VIM, and ENPP3) were significantly upregulated at the total protein level in tumor tissues compared to patient-matched NAT controls (baseline = 1) (Figure 4D). This suggests that their presentation is driven by underlying oncogenic protein overexpression coupled with active immune engagement.

Together, these findings demonstrate that multi-tier immunopeptidomics enables the high-confidence identification of functional, multi-engaged HLA I and II tumor antigens, whose robust cross-fractional presentation reflects both active local immune engagement and global oncogenic protein overexpression in RCC.

### Mechanistic Divergence Governing HLA I Alignment and HLA II Decoupling in Tumor Microenvironments

To elucidate the upstream molecular mechanisms driving the hyper-immunogenic peptide presentation observed in immune-hot tumors, we systematically crossreferenced individual immunopeptidomic throughput profiles with paired tissue-level global proteomic data (Figure 5 and Figure 6). For the HLA I stream, immune-hot tumors displayed a profoundly elevated presentation density across all target-specific, TCR-associated, and IgG-associated fractions (Figure 5A). Intriguingly, a direct comparison revealed a positive linear correlation between total HLA I presentation throughput (PSM counts) and tissue HLA I protein expression level (R = 0.883, *p* = 1.05*10^-10) across the entire cohort (Figure 5B). To decipher the pathways coordinating this synchronized presentation cascade, we performed gene set enrichment analysis (GSEA) on the tissue proteome. The antigen presentation machinery (APM) pathway emerged as an upregulated gene set, showing highly significant, uniform enrichment in both Immune-hot vs. Immune-cold (*p* = 4.855*10^-10) and Immune-hot vs. NAT (*p* = 1.29*10^-8) configurations (Figure 5C). Heatmap analysis confirmed the coordinated activation of critical node machinery (including HLA-A/B/C, B2M, and key chaperones) within the immune-hot cluster (Figure 5D). To substantiate this at individual component levels, we evaluated downstream peptide processing and transport machinery. Both TAP1 (R = 0.79, *p* = 2.4*10^-6) and TAP2 (R = 0.88, *p* = 2.7*10^-10) protein expression levels exhibited positive correlations with global HLA-I PSM counts (Figure 5E and 5F). Furthermore, STAT1, which is the master upstream transcription factor driving interferon-gamma (IFN-gamma) responsive antigen presentation was also tightly coupled with presentation output (R = 0.89, p = 1.6*10^10) (Figure 5G). Collectively, these findings delineate a highly choreographed, STAT1-mediated transcriptional program that linearly scales up HLA I structural presentation and peptide loading efficiency in immune-hot tumors.

In contrast, the regulatory landscape governing the HLA II stream proved to be substantially more complex and uncoupled (Figure 6). Although immune-hot tumors yielded elevated PSM counts across specialized fractions (Figure 6A), global HLA-II presentation throughput displayed a weak, statistically non-significant correlation with parental tissue HLA II protein abundance (R = 0.302, *p* = 0.105) (Figure 6B). This divergence indicates that surface HLA II peptide density cannot be predicted simply by total tissue protein expression. To explore this uncoupling, we dissected the HLA-II presentation cascade into functional sub-pathways via targeted GSEA (Figure 6C, D, and E). Unlike the highly orchestrated upregulation seen in HLA I, these specific HLA II sub-compartments failed to demonstrate consistent, uniform enrichment across the cohort. While core HLA II molecules and chaperone loading modules showed partial enrichment in the immune-hot vs. immune-cold comparison, key downstream operational axes, such as endolysosomal transport, lysosomal proteases, and specialized transcriptional regulators were fractured and fell short of statistical significance. Granular functional heatmap profiling further highlighted this intra-pathway heterogeneity, where lysosome processing machinery (e.g., CTSL, LGMN, CTSB) and peptide loaders exhibited discordant expression patterns across individual immune-hot and -cold specimens (Figure 6C, D, and E). Consequently, this multi-layered, asynchronous regulation of auxiliary presentation components breaks down the straightforward causal relationship between total protein expression and surface immunopeptide presentation, revealing a context-dependent regulatory mechanism to the HLA II presentation.

## DISCUSSION

In this study, we addressed two bottlenecks in clinical immunopeptidomics: the prohibitive requirement for large tissue inputs and the absence of functional, receptor-engagement contexts. By establishing a sequential immunoaffinity extraction and in vitro reconstitution platform capable of profiling from 10 mg of tissue, we systematically delineated both baseline and receptor-engaged (IgG- and TCR-bound) HLA I and II immunopeptidomes across a clinical cohort of renal cell carcinomas.

A central conceptual advance of this approach is its ability to isolate functionally relevant immunopeptide subpopulations without sacrificing analytical depth or sequence motif purity (>96% alignment for both HLA I and II). Traditional immunopeptidomic workflows predominantly capture total surface presentation, failing to distinguish passively presented self-ligands from those actively engaged by the host immune repertoire. By integrating direct native capture (nIgG/nTCR) with in vitro reconstitution (bIgG/bTCR), we demonstrate that functional receptor engagement markedly enriches for tumor-specific peptides (TSPs), achieving up to 5-fold higher TSP purity ratios compared to direct baseline capture (Figure 2D and Figure 3H). This enrichment efficiency was systematically higher in immune-hot tumors, proving that local immune infiltration naturally concentrates functionally recognized tumor epitopes.

Extending this framework to post-translationally modified (PTM) ligands revealed distinct immune subtype dependent presentation dynamics. Network analyses resolved tumor-specific phosphopeptide hubs (e.g., MUC16 and TTN), alongside N-glycan signatures such as CD68-derived glycopeptides exclusive to immune-hot tumors, reflecting heavy tumor-associated macrophage infiltration. Furthermore, examining per-sample presentation dynamics highlighted sample C3N-01648, which displayed a low global baseline HLA II peptide yield (Figure 4A) alongside an enrichment of phosphorylated species (>1,400 PSMs; Supplementary Figure 7). Integrated tissue proteomics revealed that C3N-01648 harbored high total HLA Class II protein expression together with markedly elevated levels of key endolysosomal proteases, CTSL and CTSB (Figure 6C). These results illuminate a mechanism of proteolytic over-processing: hyper-active CTSL/CTSB cleavage likely causes excessive degradation of canonical unmodified proteins into fragments unfit for HLA II loading, thereby dampening global peptide yield. Conversely, post-translational phosphorylation may confer steric or electrostatic protection against endolysosomal proteolysis, selectively preserving modified ligands for HLA II loading. Sample C3N-01648 thus exemplifies how downstream protease kinetics, rather than HLA II protein abundance alone, dictates the selectivity and composition of the presented immunopeptidome (Supplementary Figure 7). Crucially, the multi-fractional co-identification of high-priority targets (such as VIM, NNMT, TMBIM6, and ENPP3) whose parental proteins were significantly upregulated in companion total proteomics provides internal validation that these overexpressed antigens are functionally presented and actively engaged within the tumor microenvironment (Figure 4B, C, and D).

Beyond HLA peptides discovery, our integrated proteogenomic analysis uncovers a fundamental mechanistic divergence governing HLA I and II antigen presentation in primary RCC tumors (Figure 5 and 6). For HLA I, surface presentation density is tightly and linearly coupled to total tissue HLA protein abundance (R = 0.883) via a STAT1-mediated transcriptional program that uniformly scales up the entire processing and transport cascade in immune-hot tumors (Figure 5B and E). In stark contrast, HLA II presentation displays profound decoupling from parental protein abundance (R = 0.302) (Figure 6B). Targeted sub-pathway profiling revealed that while core HLA II proteins are increased in immune-hot tumors, downstream operational axes specifically endolysosomal transport and key lysosomal processing proteases (CTSL, LGMN, CTSB) exhibit heterogeneity (Figure 6D). This asynchronous regulation of auxiliary processing machinery breaks the linear causality between baseline protein expression and surface presentation output, revealing a post-transcriptional processing bottleneck governing the HLA II presentation landscape in renal cell carcinoma.

While this platform represents a substantial leap forward in low-input functional immunopeptidomics, certain limitations warrant consideration. First, while the in vitro reconstitution binding assay successfully identifies physical interactions between isolated HLA-peptide complexes and native receptor proteins (IgG/TCR), it does not directly capture the downstream signaling or functional activation kinetics of T or B cells in vivo. Future studies utilizing reporter cell lines or single-cell TCR sequencing coupled with functional activation assays will be necessary to fully map the cytotoxic or regulatory outcomes of these engagements. Second, although the current cohort provides strong statistical power to observe the stark divergence between HLA-I and HLA-II regulation across immune-hot and cold settings, expanding this sequential workflow to larger, multi-center clinical cohorts across diverse tumor histologies will further validate the generalizability of these unmutated public targets.

In conclusion, this scalable, micro-input platform bridges the gap between passive antigen presentation and active immune recognition. By capturing structural fidelity, PTM modifications, and receptor-engagement contexts from minimal clinical specimens, this framework establishes a foundation for prioritizing immunogenic neoantigens, rationalizing TCR-based T-cell therapies, and accelerating the development of TCR-like antibody therapeutics against functionally validated tumor targets.

## Methods

### Sample Collection and Clinical Cohort Description

A total of 30 clinical renal cell carcinoma (RCC) tissue specimens, comprising 18 primary RCC tumor tissues and 12 paired normal adjacent tissues (NATs), were obtained for functional immunopeptidomic profiling. Institutional review boards at each tissue source site reviewed protocols and consent documentation, in adherence to Clinical Proteomic Tumor Analysis Consortium (CPTAC) guidelines^39,40^. All tissue specimens were fresh-frozen immediately post-resection, stored at −80 °C, and maintained as cryopulverized tissue fragments before downstream lysis.

### Tissue Lysis and Protein Solubilization

Cryopulverized clinical renal cell carcinoma (RCC) tissue specimens (~10 mg) were maintained on ice and lysed by adding 120 μL of ice-cold specialized lysis buffer. The lysis buffer was prepared in a total volume of 50 mL containing 20 mM Tris-HCl (pH 8.0), 100 mM NaCl, 6 mM MgCl_2_, 1 mM EDTA, 1.5% (v/v) Triton X-100, 60 mM octyl-beta-D-glucopyranoside, 0.2 mM iodoacetamide (IAA), 1 mM phenylmethylsulfonyl fluoride (PMSF), and one tablet of EDTA-free Complete Protease Inhibitor Cocktail. The tissue-buffer mixture was subjected to complete mechanical and chemical disruption via four iterative cycles consisting of vigorous vortexing for 20 s at maximum speed followed by a 15-min incubation resting on ice at 4 °C. To digest genomic DNA and reduce lysate viscosity, 2 μL of Benzonase Nuclease was added to the mixture, followed by incubation on ice for 30 min with gentle inversion. The raw lysates were subsequently cleared by centrifugation at 15,000 × g for 20 min at 4 °C to pellet insoluble cellular debris. The clarified supernatant was immediately directed into the sequential multi-axis immunoaffinity enrichment pipeline.

### Sequential Immunoaffinity Enrichment

To isolate distinct functional immunopeptidome fractions from minimal tissue input, a continuous, sequential immunoaffinity extraction protocol was executed utilizing GammaBind Plus Sepharose beads. All micro-scale affinity captures were conducted overnight at 4 °C under constant, moderate rotation. Unbound flow-through fractions from each enrichment were systematically conserved and directed as the input material for the subsequent enrichment tier.

(1) Isolation of Endogenous IgG and IgG-Associated Peptides. Prior to incubation, 37.5 μL of GammaBind Plus Sepharose bead slurry was washed twice with 10 mM phosphate-buffered saline (PBS). The clarified tissue lysate was directly added to the pre-washed beads and incubated overnight. The incubation mixture was transferred into a microcentrifuge column fitted with a 10 μm polyethylene (PE) fritted filter and centrifuged at 1000 × g for 1 min at 4 °C. The flow-through, containing depleted lysates, was collected and reserved for downstream HLA-II and HLA-I capture. The bead matrix retained in the column was washed sequentially to eliminate non-specifically bound material using two rounds of 200 μL ice-cold complete wash buffer (20 mM Tris-HCl pH 8.0, 100 mM NaCl, 1 mM EDTA, 6 mM octyl-beta-D-glucopyranoside, and 0.2 mM IAA) followed by two rounds of 200 μL ice-cold 10 mM PBS. To isolate the native IgG-bound functional peptidome, the beads were resuspended in 100 μL of an organic elution cocktail consisting of 3% (v/v) acetonitrile (ACN) and 5% (v/v) formic acid (FA) and transferred onto a 10 kDa molecular weight cut-off (MWCO) ultrafiltration spin column. The underlying flow-through was captured. The beads were further extracted on the ultrafilter using an additional 100 μL of 3% ACN / 5% FA, followed by three consecutive washes with 100 μL of 10% (v/v) acetic acid (AcOH) at 4 °C, and a final rinse with 50 μL of 1% (v/v) FA. The pooled ultrafiltration filtrate, representing the isolated IgG-associated peptidome, was evaporated to dryness under speedvac. The remaining bead-bound IgG protein complex was neutralized with 0.2 M sodium phosphate buffer (pH 7.4; compiled from 39.2% 0.2 M NaH_2_PO_4_ and 60.8% 0.2 M Na_2_HPO_4_) and stored at 4 °C.

(2) Isolation of HLA-Class II Peptides. The depleted lysate flow-through from (1) was transferred to a clean microcentrifuge tube containing 37.5 μL of pre-washed GammaBind Plus Sepharose beads combined with 15 μg of a multiplexed HLA-II monoclonal antibody cocktail. The cocktail comprised 9 μg of clone TAL-1B5, 3 μg of clone EPR11226, and 3 μg of clone B-K27. Following overnight rotational incubation at 4 °C, the slurry was spun down at 1000 × g for 1 min at 4 °C. The secondary flow-through was retrieved and saved for (3) enrichment. The HLA-II-bound bead matrix was resuspended in 200 μL of ice-cold PBS, transferred to a 10 μm PE fritted column, and washed a total of six times to remove non-specific background matrices: three washes with 200 μL of ice-cold complete wash buffer and three washes with 200 μL of cold 10 mM PBS. Peptide-MHC complexes were dissociated by resuspending the beads in 100 μL of 3% ACN / 5% FA. The mixture was transferred to a 10 kDa MWCO ultrafiltration column to separate the low-molecular-weight HLA-II restricted peptides from the high-molecular-weight antibody-receptor complexes. The filtration was completed by sequentially washing the beads with an additional 100 μL of 3% ACN / 5% FA, three volumes of 100 μL of 10% AcOH at 4 °C, and 100 μL of 1% FA. The combined filtrate containing HLA-II peptides was dried in a speedvac. The beadanchored protein complexes were neutralized and stored at 4 °C.

(3) Isolation of HLA-Class I Peptides. The tertiary flow-through isolated from (2) was integrated with 37.5 μL of pre-washed GammaBind Plus Sepharose beads pre-bound or mixed with 15 μg of anti-human HLA-Class I monoclonal antibody (clone W6/32). After overnight capture at 4 °C, the mixture was centrifuged at 1000 × g for 1 min at 4 °C. The flow-through was meticulously harvested as the substrate for (4) enrichment. The retained beads were immediately resuspended in 100 μL of ice-cold complete wash buffer and loaded onto a pre-washed 10 μm fritted column. Stringent background clearance was performed via six fluidic washes (three cycles of 200 μL cold complete wash buffer followed by three cycles of 200 μL cold 10 mM PBS). The purified HLA-I immunopeptidome fraction was released from the matrix using 100 μL of 3% ACN / 5% FA and processed through a 10 kDa MWCO ultrafiltration spin column. The elution path was supplemented by rinsing with 100 μL of 3% ACN / 5% FA, three washes of 100 μL of 10% AcOH at 4 °C, and a final 100 μL wash of 1% FA. The collective ultrafiltration flow-through housing the HLA-I native immunopeptides was concentrated to dryness by speedvac, and the solid phase protein-bead remnants were neutralized and stored at 4 °C.

(4) Isolation of T-Cell Receptor (TCR)-Associated Peptides. In the final enrichment, the HLA-depleted flow-through from (3) was added to 37.5 μL of pre-washed GammaBind Plus Sepharose beads coupled with 15 μg of anti-TCR alpha/beta monoclonal antibody. Following overnight incubation on a rotor at 4 °C, the sample was centrifuged at 1000 × g for 1 min at 4 °C to separate the final depleted lysate. The TCR-complexed bead matrix was resuspended in 100 μL of cold complete wash buffer and isolated within a 10 μm PE fritted column. The column was washed six times (three washes with 200 μL cold complete wash buffer, followed by three washes with 200 μL cold 10 mM PBS). The TCR-associated native peptides were eluted by acid dissociation using 100 μL of 3% ACN / 5% FA and separated from the intact protein/antibody structures using a 10 kDa MWCO ultrafiltration column. The extraction was finalized with consecutive passes of 100 μL of 3% ACN / 5% FA, three volumes of 100 μL of 10% AcOH at 4 °C, and 100 μL of 1% FA through the ultrafilter. The gathered peptide filtrates evaporated to dryness using a speedvac. The remaining bead-bound protein fractions were neutralized and stored at 4 °C.

### In Vitro Reconstitution and Isolation of Receptor-Engaged HLA Peptides

To identify the specific subpopulation of HLA-I and HLA-II peptides capable of direct physical interaction with endogenous T-cell receptors (TCRs) or immunoglobulins (IgG), an in vitro reconstitution binding assay was performed. Aliquots half of the peptides obtained from the primary HLA-I and HLA-II enrichment tracks were resuspended and reconstituted in 10 mM PBS (pH 7.4). These peptide solutions were subsequently introduced to the neutralized, bead-bound intact IgG or TCR protein complexes that had been systematically recovered and stored from (1) and (4), respectively.

The peptide-protein-bead mixtures were incubated overnight at 4 °C under moderate, continuous multi-dimensional rotation to facilitate equilibrium binding. Following incubation, the slurry was transferred into a microcentrifuge column equipped with a 10 μm polyethylene (PE) fritted filter and centrifuged at 1000 × g for 1 min at 4 °C to deplete unbound or low-affinity passenger peptides. The remaining bead matrix, now hosting the reconstituted receptor-peptide complexes, was subjected to a washing regimen identical to the primary isolation axes to eliminate non-specific carryovers: three washes with 200 μL of ice-cold complete wash buffer (20 mM Tris-HCl pH 8.0, 100 mM NaCl, 1 mM EDTA, 6 mM octyl-β-D-glucopyranoside, and 0.2 mM IAA) followed by three washes with 200 μL of cold 10 mM PBS.

To capture the specific receptor-engaged peptide fractions, acid-induced dissociation was performed by resuspending the washed bead matrix in 100 μL of an elution cocktail containing 3% (v/v) ACN and 5% (v/v) FA. The resulting eluate was immediately loaded onto a 10 kDa molecular weight cut-off (MWCO) ultrafiltration spin column and centrifuged to harvest the low-molecular-weight peptides in the underlying filtrate while retaining the large macro-protein receptor structures. To ensure exhaustive recovery of the interacting ligands, the beads were sequentially rinsed with an additional 100 μL of 3% ACN / 5% FA, three iterative volumes of 100 μL of 10% (v/v) AcOH at 4 °C, and a final pass of 100 μL of 1% (v/v) FA through the ultrafilter. The ultrafiltration filtrates, containing the validated TCR-bound or IgG-bound HLA-I/II peptides, were concentrated to dryness by speedvac prior to downstream liquid chromatography-tandem mass spectrometry (LC-MS/MS) profiling.

### LC-MS/MS analysis

All mass spectrometry data were acquired using a timsTOF HT mass spectrometer (Bruker) coupled with an EvoSep One liquid chromatography system. Peptide samples were separated using Whisper Zoom 40 SPD (WZ-40 SPD) with a PepSep C18 column (15 cm × 75 μm × 1.9 μm). All LC methods employed were established protocols by Evosep^41^. MS1 spectrum was recorded across an m/z range of 100 to 1700. Ion mobility scanning was configured from 1/K0 = 1.7 to 0.6 Vs cm−2 for HLA-I or 1/K0 = 1.3 to 0.6 Vs cm−2 for HLA-II in DDA-PASEF mode. Collision energy (CE) was ramped linearly with respect to ion mobility, decreasing from 55 eV at 1/K0 = 1.6 Vs cm−2 to 10 eV at 1/K0 = 0.6 V cm−2. Polygon placement was adapted for singly charged HLA-I peptide species and as detailed in the pervious publication^33^.

### Identification of HLA and phosphorylated HLA Peptides

HLA peptide sequences were identified by querying LC-MS/MS spectra against reference datasets using FragPipe^42^ (v 24.0) using HLA and HLA-phospho workflow. The precursor and fragment ion tolerances were set at 15 ppm and 0.05 Da, respectively. For HLA peptides, database searches incorporated methionine oxidation, N-terminal acetylation, and cysteinylation as variable modifications, with the addition of S/T/Y-phosphorylation specifically for phosphopeptide analyses. Spectra were searched against a combined database of GENCODE V42 proteins and a contaminant list, alongside corresponding reversed decoy sequences. The digestion enzyme specificity was set to non-specific, with allowed peptide lengths restricted to 7 to 25 amino acids for HLA-II and 7 to 14 amino acids for HLA-I. Data processing and filtering were carried out using Philosopher^43^ (v4.5.1-RC10), featuring peptide probability modeling via PeptideProphet^44^, protein inference via ProteinProphet^45^, and internal false discovery rate (FDR) control. Peptide-spectrum matches (PSMs) were initially filtered at a 1% FDR threshold, followed by a sequential filtering step to exclude any PSMs mapped to proteins failing the 1% protein-level FDR cutoff.

### Identification of N-glycosylated HLA peptides

The raw files were executed within the FragPipe (v 24.0) using HLA-glyco workflow^27^. For glycopeptide analysis, database searches were conducted using a compilation of 707 unique glycans^46^ alongside variable modifications of methionine oxidation, N-terminal acetylation, and cysteinylation. The search space comprised GENCODE V42 proteins combined with a contaminant list and their respective reversed decoy sequences. Digestion was configured as non-specific, with peptide length boundaries set to 7–25 amino acids for HLA-II and 7–14 amino acids for HLA-I. Initial data processing was performed using Philosopher^43^ (v4.5.1-RC10), utilizing PeptideProphet^44^ for probability modeling and ProteinProphet^45^ for protein inference. To control error rates, a modified group-specific FDR strategy was implemented in Philosopher, applying independent 1% FDR thresholds to non-glycosylated and glycosylated PSMs. A subsequent filtering filter removed any PSMs associated with proteins failing the 1% protein-level FDR. Finally, glycan localization and assignment were executed in PTM-Shepherd utilizing default N-glycan parameters and database entries, coupled with a 0.05 glycan *q*-value cutoff.

### Motif Clustering and Deconvolution

To identify and characterize shared binding motifs among the identified peptide sequences, unsupervised clustering was performed using the GibbsCluster^47,48^ (version 2.0). Peptide sequences of specific lengths were grouped based on their sequence similarity and predicted binding specificities using the default parameters provided by the tool.

### Subtractive Bioinformatic Filtering and TSP Purity Ratio Calculation

To isolate tumor-specific peptides (TSPs), immunopeptidomes generated from primary RCC tumor tissues were filtered against patient-matched normal adjacent tissue (NAT) controls. Any unique peptide sequence identified in NAT samples across the cohort was subtracted from the tumor peptide matrix. To account for variations in baseline presentation recovery across samples, normalized TSP purity ratios were calculated using the formula:

$$\text{Normalized TSP Purity Ratio}=\frac{\text{Fractional TSP Count}}{\text{Fractional HLA Yield}}\times \left(\frac{\text{Total HLA Yield}}{\text{Fractional HLA Yield}}\right)$$

Purity ratios were stratified across immune-hot and immune-cold tumor microenvironment phenotypes as defined by immune cell infiltration^39,40^.

### Gene Set Enrichment Analysis (GSEA) and Multi-Omic Integration

Global protein intensity matrices were normalized by median centering^49^. Differential protein expression analysis between immune subtypes (Immune-hot vs. Immune-cold; Immune-hot vs. NAT) was conducted using Moderated t-tests in the limma R package. Pre-ranked Gene Set Enrichment Analysis (GSEA) was performed using the clusterProfiler^50^ package (v4.10.1) against MSigDB^51^ canonical pathways and custom antigen presentation machinery (APM) gene sets. Normalized Enrichment Scores (NES) and adjusted *p*-values (Benjamini-Hochberg) were calculated using 10,000 permutations. Linear regression and correlation analyses (Pearson r) comparing global protein expression to total immunopeptidome throughput (PSM counts) were executed in R (v4.3.3).

### Statistical Analysis

Quantitative differences between two groups were evaluated using two-tailed Student’s t-tests or non-parametric Wilcoxon rank-sum tests where appropriate. Multigroup comparisons were evaluated using one-way ANOVA followed by Tukey’s posthoc test. Statistical significance was designated as * p < 0.05, ** p < 0.01, and *** p < 0.001. All statistical calculations and data visualizations were executed within R (v4.3.3).

### Supplementary Material

Supporting Information: Supplementary Figure 1. Schematic workflow of the functional immunopeptidomics for receptor-associated antigens platform. Supplementary Figure 2. UpSet intersection matrix delineating the cohort-wide cross-patient sharing profiles and exclusive partitioning of HLA-II restricted TSPs. Supplementary Figure 3. UpSet plot analysis of tumor-specific phosphorylated peptides and their corresponding source proteins. Supplementary Figure 4. Network visualization of shared tumor-specific phosphorylated peptides and source proteins. Supplementary Figure 5. Representative MS/MS spectra of identified N-glycopeptides. Supplementary Figure 6. UpSet intersection matrix delineates the cohort-wide cross-patient sharing profiles and exclusive partitioning of HLA-I restricted TSPs. Supplementary Figure 7. Distribution of HLA II-restricted phosphopeptide PSMs across RCC tumor samples.

This is a list of supplementary files associated with this preprint. Click to download.

- SupplementaryFigure.pdf

## Figures and Tables

**Figure 1.**
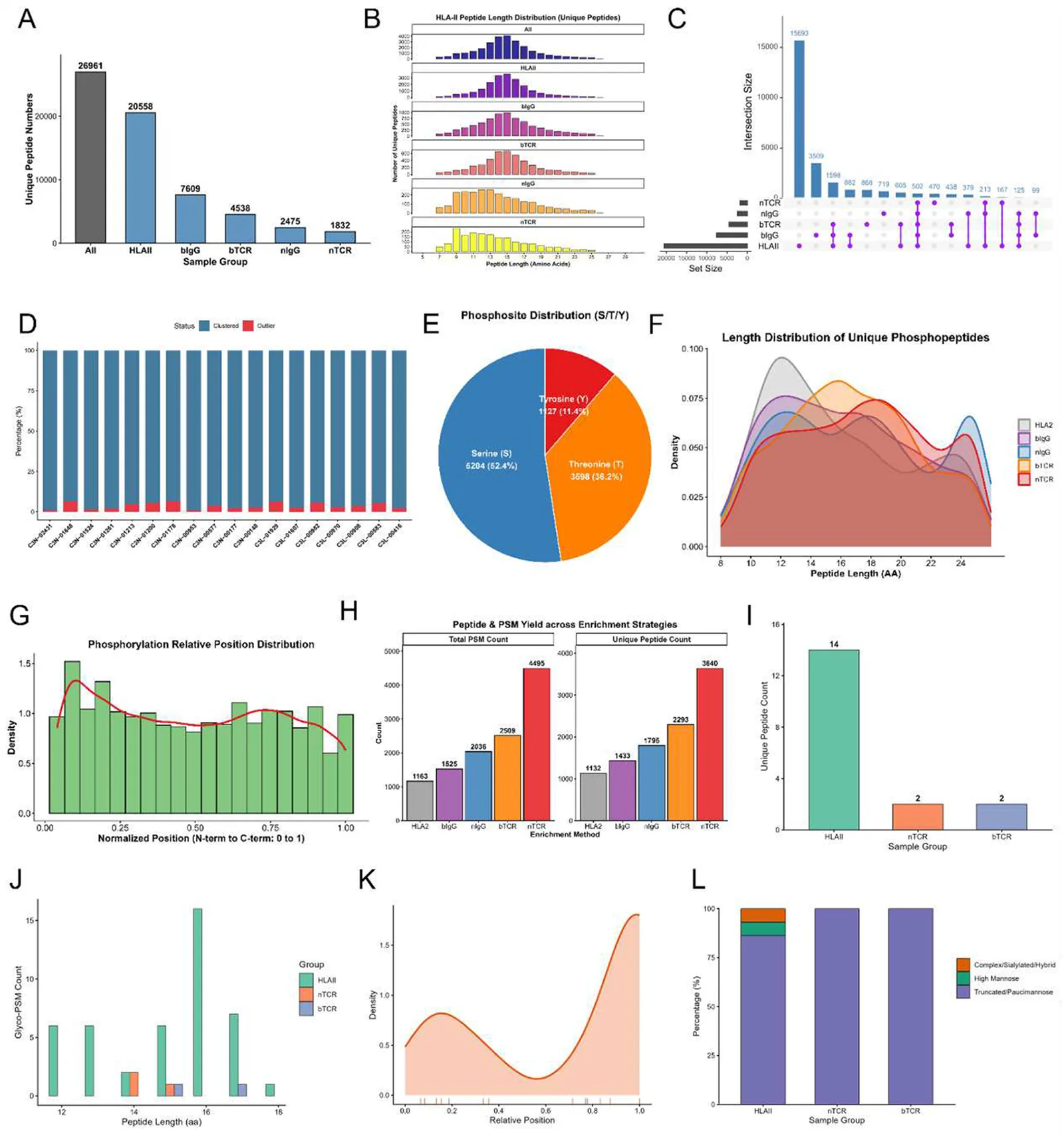
Profiling of Functional HLA II-associated global, phosphorylated, and N-glycosylated immunopeptides. (A) Total unique peptide numbers identified across the global immunopeptidome and functional enrichment sample groups. (B) Peptide length distribution profiles of unique HLA II peptides across individual enrichment sample groups. (C) UpSet intersection matrix showing the overlap and sharing patterns of unique peptides identified across distinct enrichment sample groups. (D) Samp-lelevel status clustering evaluation verifying data stability and quality across the cohort. (E) Relative abundance proportions of phosphorylated amino acid residues (Serine, Threonine, and Tyrosine) identified within the phosphorylation immunopeptidomic dataset. (F) Length distribution density profiles of unique phosphopeptides recovered across different enrichment sample groups. (G) Spatial relative position distribution of phosphorylation modifications mapped from the N-terminus to the C-terminus. (H) Total peptide-spectrum match (PSM) counts, and unique peptide yields across distinct enrichment methods. (I) Unique glycopeptide counts identified across HLA II and TCR-associated sample groups. (J) Glycopeptide-PSM counts distributions grouped by peptide length and enrichment sample group. (K) Relative position distribution profile of N-glycosylation sites along the peptide sequence. (L) Structural glycoform subtype proportions (Complex/Sialylated/Hybrid, High Mannose, and Truncated/Paucimannose) across captured sample groups.

**Figure 2.**
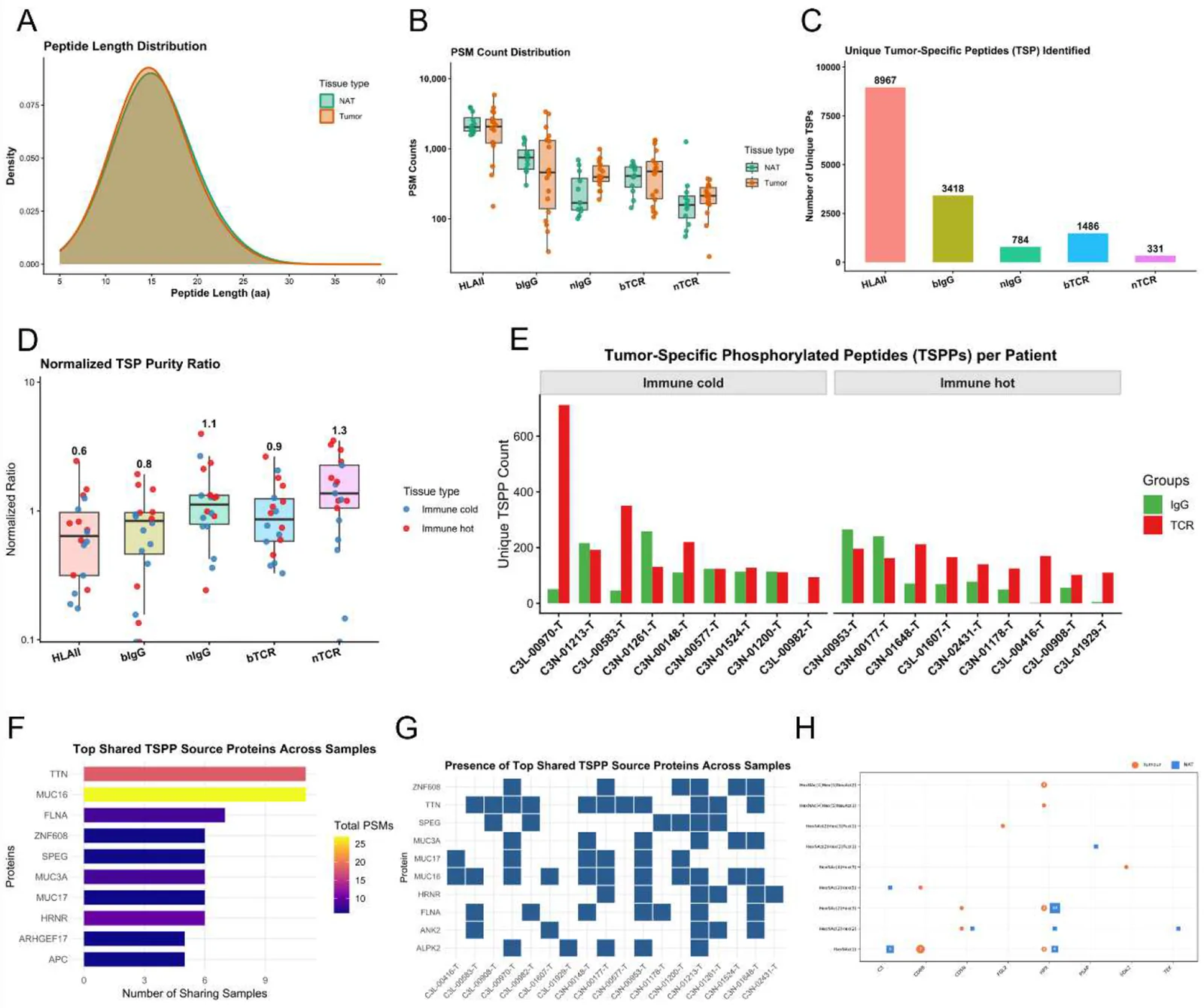
Characterization of HLA II-restricted tumor-specific peptides, phosphorylated peptides, and N-glycosylated peptides in renal cell carcinoma. (A) Peptide length distribution profiles of HLA II-restricted peptides comparing tumor tissues versus normal adjacent tissues (NAT). (B) PSM count distributions of HLA II-restricted peptides per sample categorized by enrichment sample group and tissue status on a log scale. (C) Total counts of unique HLA II-restricted tumor-specific peptides (TSPs) across enrichment sample groups. (D) Normalized HLA II-restricted TSP purity ratios per patient stratified by immune subtype (immune-hot vs. immune-cold) across enrichment sample groups. (E) Counts of HLA II-restricted unique tumor-specific phosphorylated peptides (TSPPs) per patient and stratified by immune subtypes and enrichment sample groups. (F) Top shared HLA II-restricted TSPP source proteins across the RCC patient cohort ranked by the number of sharing patients and cumulative PSM frequency. (G) Binary presence-absence heatmap tracking the distribution of top shared HLA II-restricted TSPP source proteins across individual patient specimens. (H) PSM counts of identified HLA II-restricted N-glycosylated peptides mapped across source proteins and colored by tissue types.

**Figure 3.**
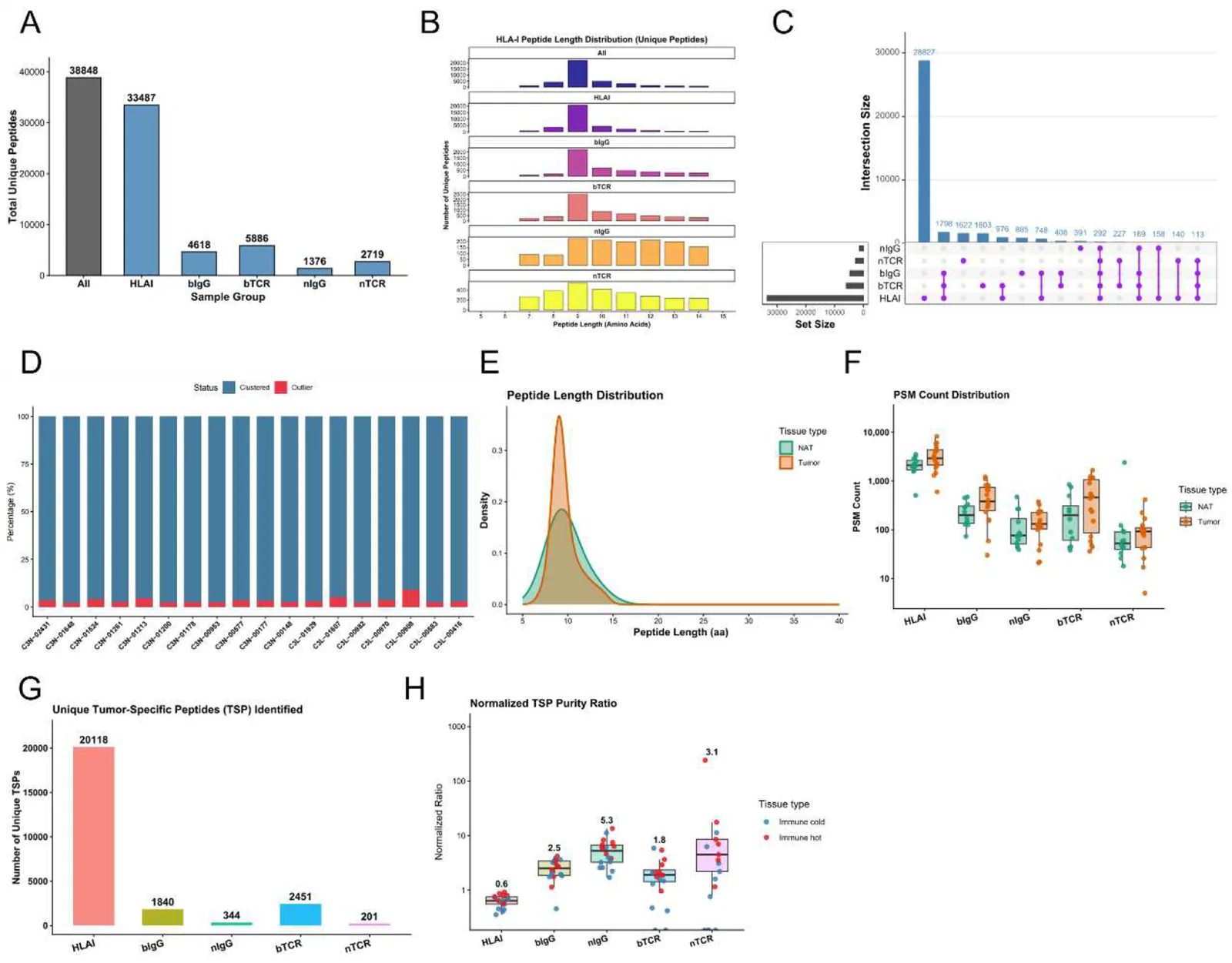
Functional immunopeptidomic profile performance of HLA I-restricted peptides (A) Total and unique peptide counts recovered across the specific enrichment sample groups. (B) Detailed peptide length distribution profiles of unique HLA I peptides across all enrichment sample groups. (C) UpSet intersection matrix illustrating the intersection sizes and overlap of unique peptide repertoires among HLA I and associated enrichment sample groups. (D) Percentage distribution of clustered versus outlier status across individual samples. (E) Comparative peptide length distribution profile between tumor tissues and normal adjacent tissues (NAT). (F) PSM count distributions per sample evaluated across enrichment sample groups and tissue types. (G) Counts of unique tumor-specific antigens identified in each enrichment sample group. (H) Normalized TSP purity ratios per sample stratified by tissue types and enrichment sample groups.

**Figure 4.**
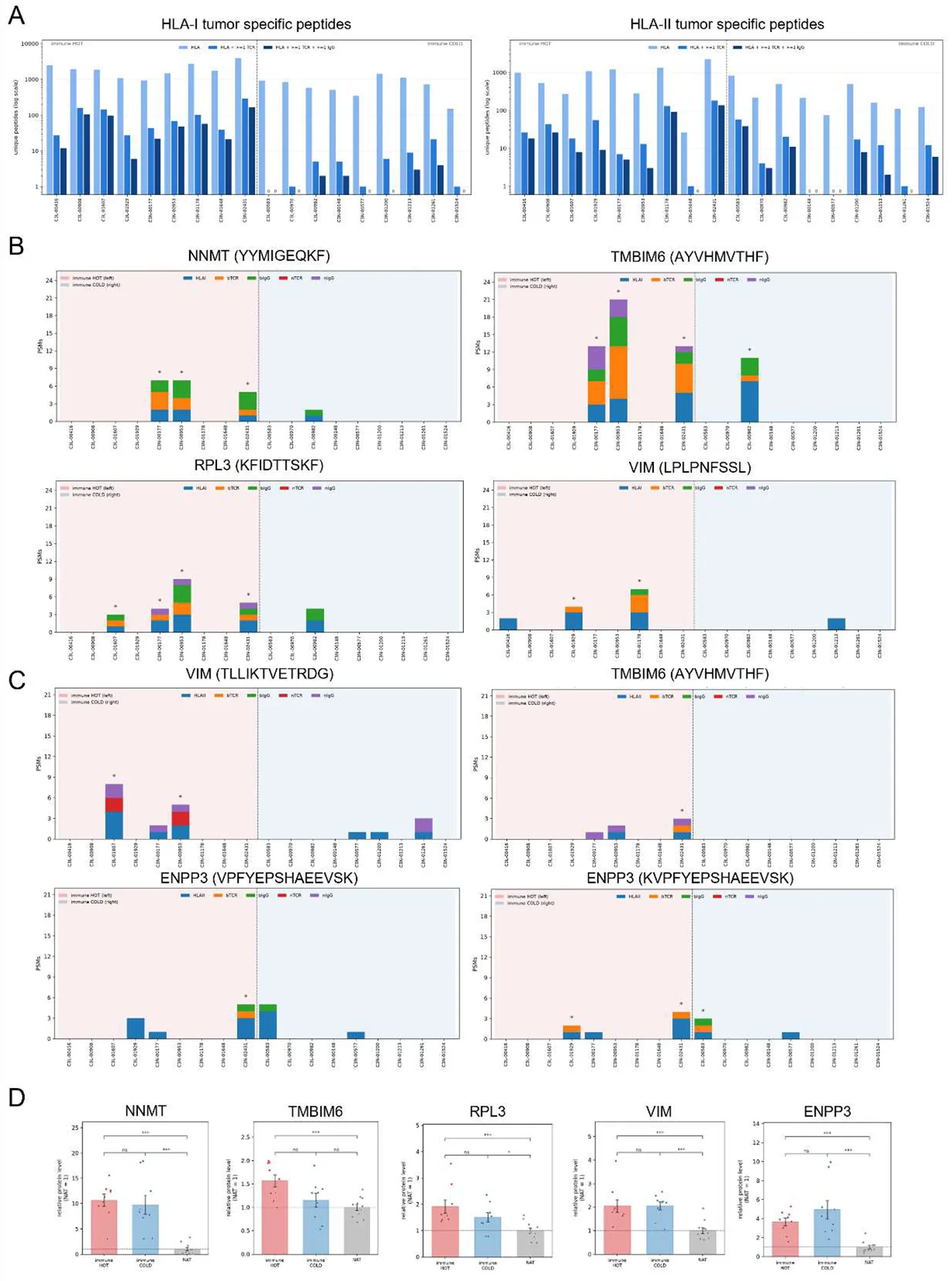
Semi-quantitative of functional tumor-specific immunopeptides. (A) Repertoire-wide analytical throughput representing unique peptide counts (log scale) of tumor-specific HLA I (left) and HLA II (right) peptides distributed across the samples, stratified into immune-hot and immune-cold immune subtypes, and separated by a vertical dashed line with stacked bar shades designating co-identification conditions (baseline HLA alone, HLA + TCR, and HLA + TCR + IgG). (B and C) Granular multi-fractional presentation profiles (Y-axis: PSM counts) of shared, tumor-specific peptides capturing for HLA I (B: NNMT, TMBIM6, RPL3, VIM) and HLA II (C: VIM, ENPP3), indexed by parental source proteins and specific amino acid sequences, with shaded backgrounds distinguishing immune subtypes (immune-hot in light pink and immune-cold in light blue) and asterisks (*) marking patient samples where the TCR target was identified. (D) Relative protein expression levels of corresponding source genes (NNMT, TMBIM6, RPL3, VIM, ENPP3) across immune-hot tumor, immune-cold tumor, and normal adjacent tissue (NAT, baseline set to 1) groups, validating tumor-specific upregulation at the global proteomic level.

**Figure 5.**
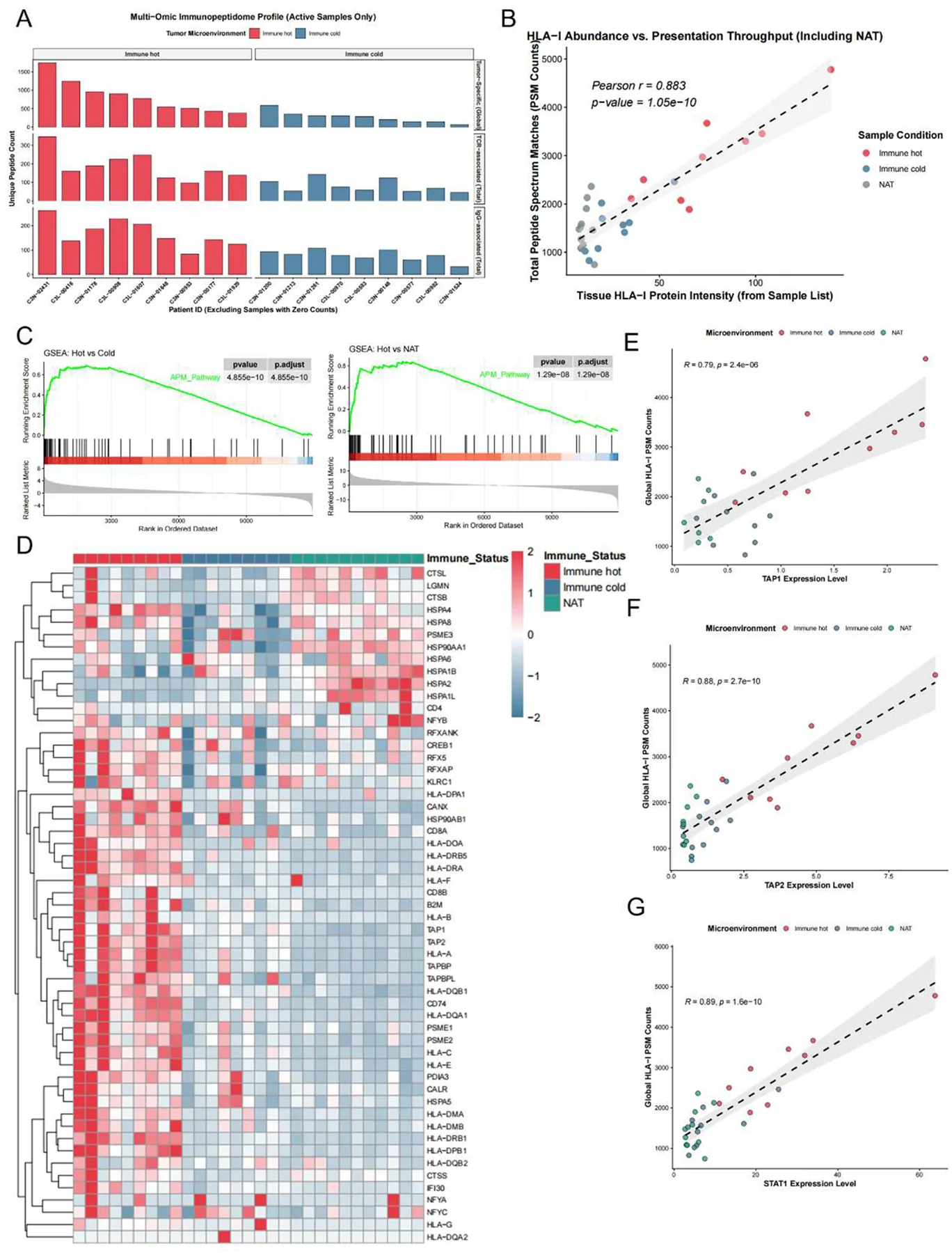
Coordinated STAT1-dependent activation of the antigen presentation machinery drives HLA class I presentation throughput. (A) Immunopeptidomic throughput profiles restricted to active patient samples (Y-axis: unique peptide counts), stratified into immune-hot (red) and immune-cold (blue) microenvironments across tumor-specific, TCR-associated, and IgG-associated fractions. (B) Scatter plot illustrating a strong positive correlation between tissue-level HLA-I protein intensity (X-axis) and global HLA-I presentation throughput (Y-axis, total PSM counts) across immune-hot tumors (red), immune-cold tumors (blue), and NAT controls (grey). Pearson correlation coefficient (r) and log-transformed *p*-value are indicated. (C) Gene Set Enrichment Analysis (GSEA) profiles of the classical Antigen Presentation Machinery (APM) pathway, demonstrating prominent positive enrichment in Immune-hot vs. COLD (left) and Immune-hot vs. NAT (right) proteomic comparisons. Exact *p*-values and adjusted *p*-values are provided inline. (D) Supervised hierarchical clustering heatmap depicting the relative tissue-level expression of core APM pathway components across individual patient samples sorted by their microenvironmental immune status. (E–G) Linear regression analyses mapping global HLA-I presentation throughput (PSM counts) against individual protein expression levels of key peptide transporters TAP1 (E), TAP2 (F), and the upstream regulatory driver STAT1 (G). Spearman rank correlation coefficients (R) and *p*-values are indicated.

**Figure 6.**
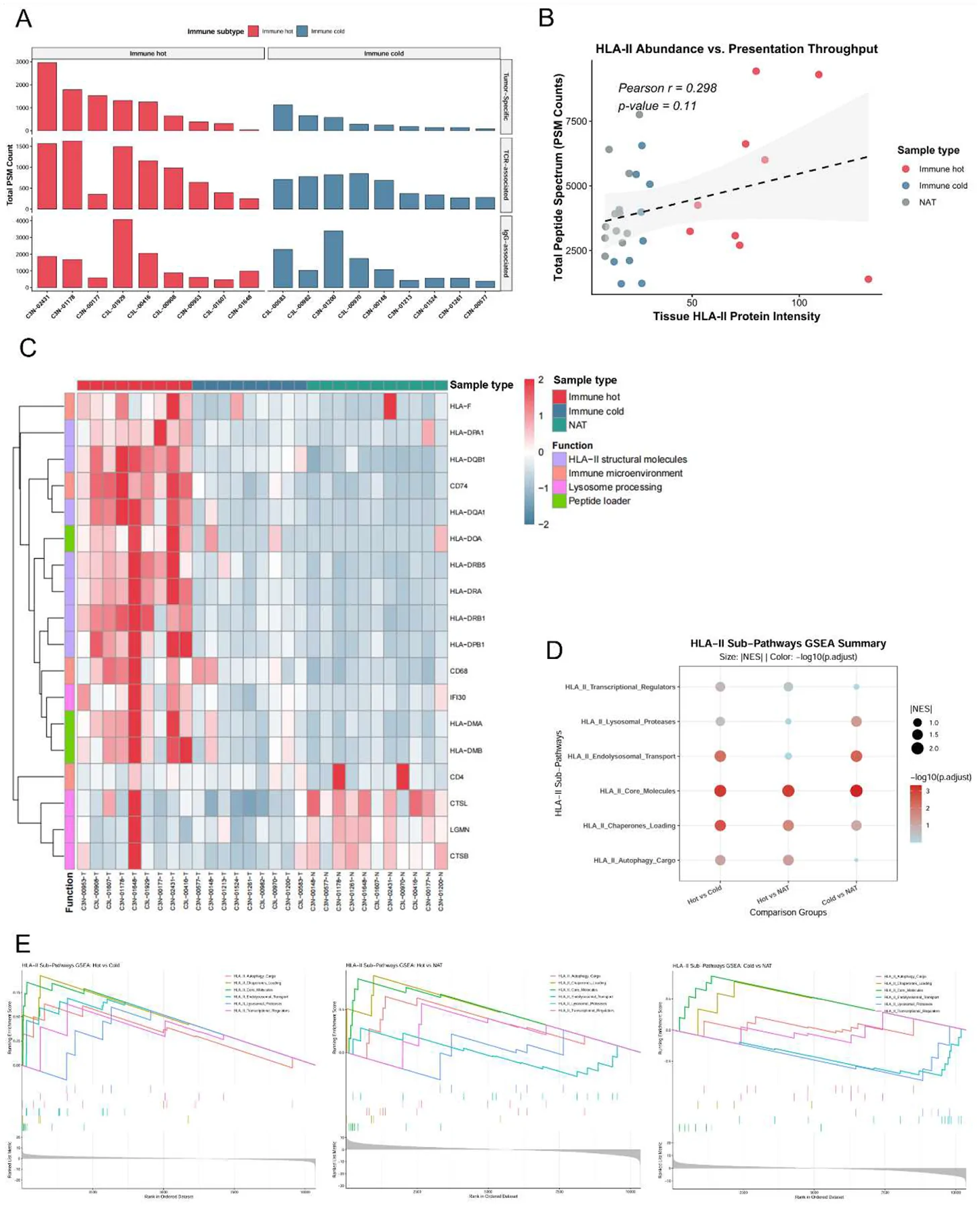
Transcriptional regulation and pathway enrichment profiling of the HLA class II antigen presentation machinery across the tumor immune subtypes. (A) HLA II immunopeptidomic PSM counts stratified into immune-hot (red) and immune-cold (blue) tumor immune subtypes across tumor-specific, TCR-associated, and IgG-associated fractions. (B) Scatter plot evaluating the correlation between tissue-level HLA-II protein intensity and global HLA-II presentation throughput (total PSM counts) across patient and control groups, with Pearson *r* and *p*-values provided. (C) Supervised hierarchical clustering heatmap displaying the relative tissue expression profiles of core HLA class II structural molecules, microenvironment factors, lysosomal processing enzymes, and peptide loaders across immune-stratified samples. (D) Summary bubble plot of HLA class II sub-pathway GSEA results showing normalized enrichment scores (NES) and statistical significance (-log10 adjusted p-value) across pairwise comparison groups (Hot vs. Cold, Hot vs. NAT, Cold vs. NAT). (E) Detailed GSEA enrichment curves tracking distinct functional sub-pathways of the HLA class II antigen presentation network across comparison phenotypes.

## Data Availability

The mass spectrometry proteomics data have been deposited to the ProteomeXchange Consortium via the PRIDE^52^ partner repository with the data set identifier PXD082540. Reviewer could log in to the PRIDE website using the following details: Project accession: PXD082540, Token: bsbv7n4wfxwL
